# Associations between endogenous sex hormone levels and adipokine levels in the Multi-Ethnic Study of Atherosclerosis

**DOI:** 10.3389/fcvm.2022.1062460

**Published:** 2023-01-13

**Authors:** Bhavya Varma, Oluseye Ogunmoroti, Chiadi E. Ndumele, Brigitte Kazzi, Carla P. Rodriquez, Olatokunbo Osibogun, Matthew A. Allison, Alain G. Bertoni, Erin D. Michos

**Affiliations:** ^1^Ciccarone Center for the Prevention of Cardiovascular Disease, Johns Hopkins School of Medicine, Baltimore, MD, United States; ^2^Department of Epidemiology, Robert Stempel College of Public Health and Social Work, Florida International University, Miami, FL, United States; ^3^Department of Family Medicine, University of California, San Diego, San Diego, CA, United States; ^4^Department of Epidemiology and Prevention, Wake Forest School of Medicine, Winston-Salem, NC, United States

**Keywords:** sex hormones, adipokines, biomarkers, cardiovascular disease, visceral fat

## Abstract

**Background:**

Differences in sex hormone levels contribute to differences in cardiovascular disease (CVD) risk. Adipokines play a role in cardiometabolic pathways and have differing associations with CVD. Adipokine levels differ by sex; however, the association between sex hormone profiles and adipokines is not well established. We hypothesized that a more androgenic sex hormone profile would be associated with higher leptin and resistin and lower adiponectin levels among postmenopausal women, with the opposite associations in men.

**Methods:**

We performed an analysis of 1,811 adults in the Multi-Ethnic Study of Atherosclerosis who had both sex hormones and adipokines measured an average of 2.6 years apart. Sex hormones [Testosterone (T), estradiol (E2), sex hormone binding globulin (SHBG), and dehydroepiandrosterone (DHEA)] were measured at exam 1; free T was estimated. Serum adipokines (leptin, resistin, adiponectin) were measured at exams 2 or 3. We used multivariable linear regression to examine the cross-sectional associations between sex hormones and adipokines.

**Results:**

The mean (SD) age was 63 (10) years, 48% were women; 59% non-White participants. For leptin, after adjusting for demographics only, higher free T and lower SHBG, were associated with higher leptin in women; this association was attenuated after further covariate adjustment. However in men, higher free T and lower SHBG were associated with greater leptin levels in fully adjusted models. For adiponectin, lower free T and higher SHBG were associated with greater adiponectin in both women and men after adjustment for CVD risk factors. For resistin, no significant association was found women, but an inverse association with total T and bioT was seen in men.

**Conclusion:**

Overall, these results further suggest a more androgenic sex profile (higher free T and lower SHBG) is associated with a less favorable adipokine pattern. These findings may provide mechanistic insight into the interplay between sex hormones, adipokines, and CVD risk.

## Introduction

Sex differences exist in the risk for cardiometabolic diseases (1), which may, in part, be due to biological differences in sex hormone levels between the sexes. While the effects of exogenous hormone treatment remains controversial, multiple studies have suggested a higher age-adjusted incidence of cardiovascular disease (CVD) among men (compared to women) and an increased CVD risk in post-menopausal women compared to similarly aged pre-menopausal women, which implicate an adverse effect of endogenous androgens on the development of CVD (2–5). A prior study from the Multi-Ethnic Study of Atherosclerosis (MESA) found that a more androgenic (“male-like”) sex hormone pattern [i.e., higher testosterone to estradiol (T/E2) ratio] among post-menopausal women was associated with increased rates of CVD, coronary heart disease (CHD), and heart failure (6). Other studies have linked higher androgen levels (free T) in post-menopausal women with endothelial dysfunction, subclinical atherosclerosis, adverse cardiac remodeling, aortic stiffness, and visceral adiposity (7–11). In contrast to women where higher androgen levels have been associated with a worse cardiometabolic profile, the opposite pattern has been noted in men. Rather, low testosterone levels in men have been linked to insulin resistance, diabetes, visceral adiposity, and CVD risk (11–16).

Men have a greater prevalence of diabetes than women; however, diabetes confers a greater relative risk of CHD and heart failure in women compared to men (17, 18). Other work from MESA has found that in women greater androgen levels were associated with a more insulin resistant phenotype (19), whereas the opposite association was seen in men (13). This parallels patterns seen with adiposity, with higher bioavailable T (bio T) being associated with greater visceral fat in women, with the reverse association in men (11). Additionally, studies have shown that lower levels of sex-hormone binding globulin (SHBG) in women have been associated with central adiposity, diabetes, and CVD risk (20–23).

Obesity may attenuate the “female-advantage” of sex differences in CVD. Adipose tissue, particularly in the visceral space, is associated with an increased risk of CVD (24–28). This fat tissue releases hormones called adipokines, which play a role in metabolism and inflammation (29, 30). Adipokines, including adiponectin, leptin, and resistin, have differing relationships with CVD risk. While adiponectin is thought to be protective against CVD, leptin and resistin are associated with increased CVD risk (31–34). Adipokine levels differ by sex (35, 36); however, the relationship of sex hormones and adipokine levels has not been well-established.

Prior studies in MESA have looked at associations between both sex hormone levels with CVD risk and adipokine levels with CVD risk (6, 32, 33). However, the association between sex hormones and adipokine levels with each other has not been examined. Therefore, we investigated the relationship between sex hormone profiles and adipokine levels in men and post-menopausal women, to provide mechanistic insight into their relationship with increased CVD risk. We hypothesized that a more androgenic sex hormone pattern would be associated with an adverse adipokine profile in women, with an opposite relationship seen in men.

## Materials and methods

### Study population

Between the years of 2000–2002, the MESA enrolled 6,814 men and women aged 45–84 years who were free of clinical CVD (37). Participants were recruited from 6 United States regions and 4 self-reported racial/ethnic groups (Black, Hispanic, Chinese, and non-Hispanic White individuals). MESA activities were approved by the institutional review boards at all field centers, and participants provided written informed consent. This study design has been previously described in detail (37).

**FIGURE 1:**
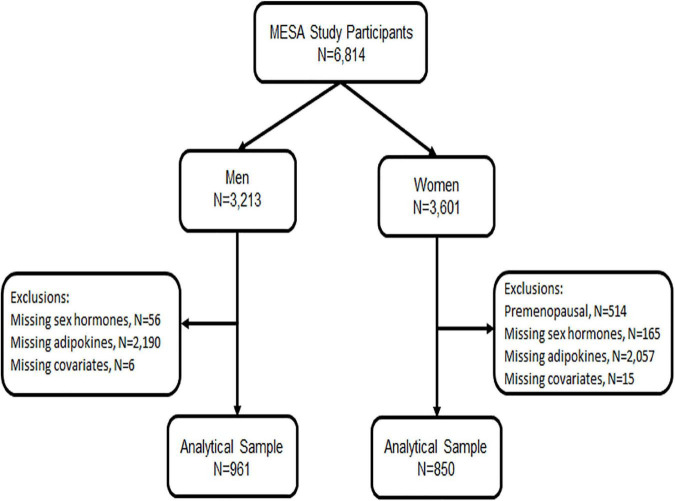
Study flow chart.

For the analysis presented below, we included all men and post-menopausal women who had endogenous sex hormone levels measured at baseline exam 1 (2000–2002) and who had adipokine levels measured at either exam 2 or 3 (2002–2005), randomly assigned, as part of an ancillary study (*n* = 1,811). We excluded pre-menopausal women given their small sample size and due to differential sex hormone distribution by menopause status (Figure 1). Post-menopausal status was determined using a previously published algorithm in MESA which included self-report, history of prior oophorectomy, and/or age > 55 years (8, 38).

### Sex hormone measurement

In an ancillary study in MESA, sex hormones were measured from fasting blood samples that were collected and stored at the time of exam 1. Serum hormone levels were measured at the Steroid Hormone Laboratory at the University of Massachusetts Medical Center (Worcester, MA). Estradiol was measured using an ultrasensitive radioimmunoassay kit (Diagnostic System Laboratories, Webster, TX). Testosterone and dehydroepiandrosterone (DHEA) were measured using radioimmunoassay kits and SHBG was measured using chemiluminescence enzyme immunometric assay using Immulite kits (Diagnostic Products Corporation, Los Angeles, CA). Bio T and free T were calculated using the total T and SHBG, as described by Sodergard (39).

### Adipokine measurement

A random subset of all MESA participants (*n* = 1,970) underwent abdominal computed tomography (CT) scans at either exam 2 or exam 3 (randomly assigned) to measure abdominal aortic calcium (40). In a subsequent ancillary study related to body composition, adipokine levels were measured among these same participants from stored frozen serum samples obtained at the respective exams (2 or 3) of their CT scan (30). The adipokines (adiponectin, leptin, and resistin) were analyzed using a Bio-Rad Luminex flow cytometry (Millepore, Billerica, MA) at the Laboratory for Clinical Biochemistry Research (University of Vermont, Burlington, VT), as previously reported (31, 32). The coefficients of variation for these assays ranged from 6 to 13%.

### Covariate measurement

For this analysis, we included covariates from exam 1, the time of the sex hormone level measurement including: age, race/ethnicity, sex, smoking status (never, former, current), body mass index (BMI), highest education level achieved, physical activity (moderate + vigorous in METS-min-week), current use of hormone therapy for women, total cholesterol, high density lipoprotein cholesterol (HDL-C), lipid-lowering medication, systolic blood pressure, use of anti-hypertensive medication, diabetes status (normoglycemia being fasting glucose of 99 mg/dL or less, impaired fasting glucose 100–125 mg/dL, and diabetes ≥ 126 mg/dL or the use of diabetes medications), and estimated glomerular filtration rate (eGFR). Using the abdominal CT data acquired as part of the aforementioned ancillary study at exam 2 or 3, visceral adipose tissue (VAT) was measured as the total adipose tissue in the abdominal cavity and subcutaneous adipose tissue (SAT) measured as the total adipose tissue outside of the abdominal cavity but not within muscle tissue. VAT and SAT were measured from the average of 2 CT slices obtained at L2–L3 and adjusted for height, as previously performed (30).

### Statistical analysis

Sex hormone levels were the exposure (independent) variable for this analysis, including total T, bio T, free T, estradiol, DHEA, SHBG, and the ratio of T/E2. Adipokine levels were the outcome (dependent) variables for this analysis, including leptin, resistin, and adiponectin.

We used a cross-sectional design as both sex hormones and adipokines were each only measured once in MESA, although it should be noted that measurements were non-concurrent with sex hormones being from the baseline exam and adipokines from exams 2 or 3, with average follow-up being 2.6 years from baseline exam. As sex hormone profiles differ by sex and are not over-lapping, all analyses were stratified separately by sex. We used descriptive statistics to report the baseline characteristics of the study population by sex, with continuous variables reported as means with their standard deviation (SD) or medians with interquartile range (IQR) for skewed distributions and categorical variables as frequencies with percentages.

We examined the unadjusted Pearson’s correlations (r) between sex hormones and adipokines. We used multivariable linear regression to examine the separate associations of the endogenous sex hormone levels of total T, bio T, free T, estradiol, DHEA, SHBG, and T/E2 ratio with adipokine levels (leptin, resistin, and adiponectin) separately. As some of the sex hormone levels and adipokines were not normally distributed, they were natural log-transformed for analysis, and then we exponentiated the beta-coefficients from the regression models to reflect their geometric mean and presented as percent differences. The sex hormones were compared continuously per 1SD increment. *A priori*, we decided to focus on free T and SHBG as our primary sex hormones of interest, as these have been shown to be most associated with subclinical CVD in prior MESA studies (7–10), but we report the findings for all sex hormones for comparison.

For all analyses, we used progressively adjusted regression models. Model 1 adjusted for age, race/ethnicity and study center. Model 2a included model 1 variables as well as lifestyle and behavioral factors such as education, physical activity, smoking status, and BMI. For women, this model was also adjusted for the use of hormone therapy. Model 2b included model 2a variables plus VAT and SAT. Model 3 included model 2 variables and CVD risk factors and medications such as systolic blood pressure, use of anti-hypertensive medication, total cholesterol, HDL-C, use of lipid-lowing medication, diabetes mellitus status, and eGFR.

In supplemental analysis, we examined for interaction by BMI (<30 vs. ≥ 30 kg/m^2^) and stratified results if a significant interaction was found.

Two-sided *P*-values of < 0.05 were considered to be statistically significant, including for interaction terms. We used STATA version 15.0 [StataCorp. (41). Stata Statistical Software: Release 15, College Station, TX, StataCorp LLC] for performing the analyses.

## Results

### Characteristics of study population

**FIGURE 2:**
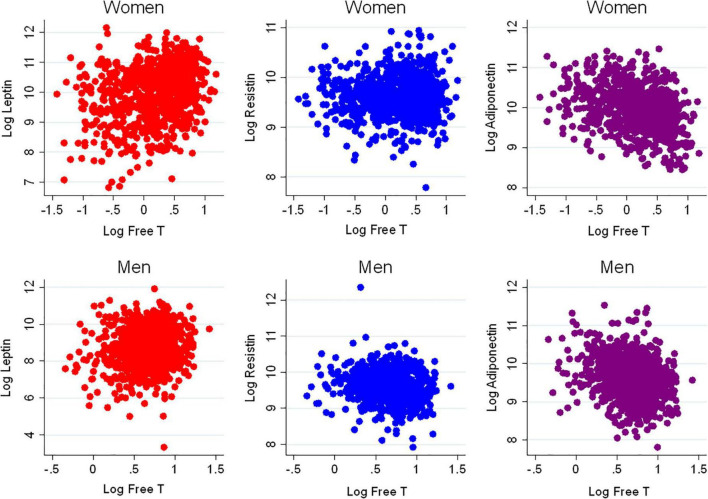
Scatterplots of adipokines vs. free testosterone. T, Testosterone.

**FIGURE 3:**
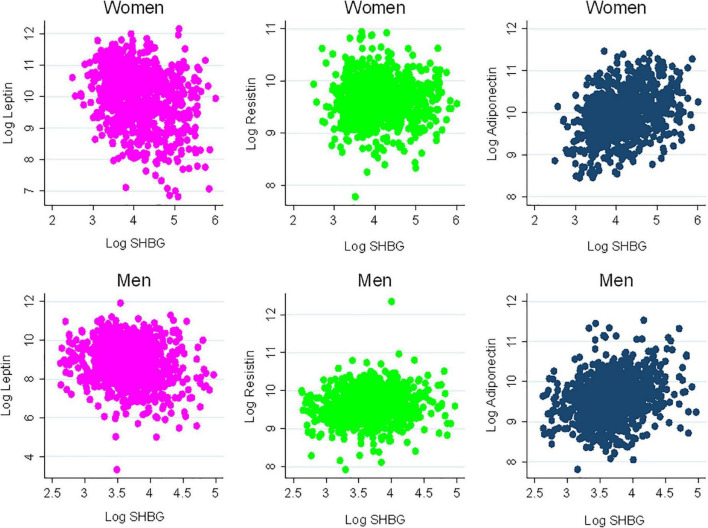
Scatterplots of adipokines vs. sex hormone binding globulin. SHBG, sex hormone binding globulin.

There were 1,811 participants with endogenous sex hormone levels and adipokine levels measured that were included in the analysis (Table 1). The mean (SD) age of participants was 63 (10) years; 47% were postmenopausal women. There were 41% White, 13% Chinese, 20% Black, and 26% Hispanic adults. Half the participants were never smokers. The average BMI was 28 (5) kg/m^2^. In terms of medication use, 37% of participants were on antihypertensives, 17% were on lipid-lowering medications, and 35% of women were currently using hormone therapy. As anticipated, men had higher T levels, as well as E2, than post-menopausal women, whereas women have higher SHBG levels. Women had higher leptin and adiponectin levels than men, with similar levels of resistin. The scatterplot of the unadjusted adipokine levels by free testosterone and SHBG, stratified by sex, are shown in Figures 2, 3, respectively.

**TABLE 1:** Characteristics of study participants.

	Total, *N* = 1,811	Women, *n* = 850	Men, *n* = 961
Leptin, ng/mL	12.8 (5.4, 27.8)	25.0 (12.6, 42.9)	7.0 (3.3, 14.3)
Resistin, ng/mL	15.1 (11.9, 19.1)	15.4 (11.9, 19.5)	14.8 (11.9, 18.7)
Adiponectin, mcg/mL	17.4 (11.8, 26.2)	21.9 (14.8, 31.5)	14.5 (10.1, 21.1)
Total T, nmoI/L	8.1 (0.9, 14.7)	0.9 (0.6, 1.3)	14.3 (11.5, 17.8)
Free T, percent	1.7 (1.3, 2.1)	1.3 (0.9, 1.7)	2.0 (1.7, 2.4)
Bio T, nmoI/L	2.9 (0.2, 5.5)	0.2 (0.1, 0.3)	5.3 (4.3, 6.6)
Estradiol, nmoI/L	0.10 (0.07, 0.14)	0.07 (0.05, 0.16)	0.11 (0.09, 0.14)
SHBG, nmoI/L	45.9 (33.6, 66.3)	58.4 (39.3, 95.9)	40.2 (31.6, 51.5)
DHEA, nmoI/L	11.5 (8.3, 16.1)	10.5 (7.1, 14.9)	12.5 (9.2, 16.9)
Total T: Estradiol ratio	68.8 (12.8, 133.8)	12.3 (4.9, 22.1)	128.8 (97.2, 169.8)
Age, years	63 (10)	64 (9)	62 (10)
**Race/Ethnicity**			
White	736 (41%)	332 (39%)	404 (42%)
Chinese-American	242 (13%)	111 (13%)	131 (14%)
Black	359 (20%)	186 (22%)	173 (18%)
Hispanic	474 (26%)	221 (26%)	253 (26%)
**Education**			
≥ Bachelor’s degree	654 (36%)	251 (30%)	403 (42%)
<Bachelor’s degree	1,157 (64%)	599 (70%)	558 (58%)
**Smoking status**			
Never	905 (50%)	499 (59%)	406 (42%)
Former	682 (38%)	258 (30%)	424 (44%)
Current	224 (12%)	93 (11%)	131 (14%)
BMI, kg/m^2^	28 (5)	28 (6)	28 (4)
Physical activity, MET-minute/week	4,050 (2,100, 7,530)	3,783 (1,965, 6,645)	4,410 (2,205, 8,693)
Systolic blood pressure, mmHg	127 (22)	129 (24)	126 (20)
Total cholesterol, mg/dL	196 (34)	202 (34)	191 (34)
HDL-C, mg/dL	51 (15)	57 (16)	45 (12)
Use of antihypertensive medication	664 (37%)	336 (40%)	328 (34%
Use of lipid-lowering medication	299 (17%)	162 (19%)	137 (14%)
Current use of HT*	−	294 (35%)	−
SAT, cm^2^	142 (101, 202)	166 (116, 225)	126 (92, 176)
VAT, cm^2^	154 (96, 227)	115 (71, 171)	201 (139, 264)
Diabetes	212 (12%)	89 (10%)	123 (13%)
eGFR, mL/min/1.73 m^2^	73 (15)	71 (15)	75 (15)

Bio, bioavailable; BMI, body mass index; DHEA, dehydroepiandrosterone; eGFR, estimated glomerular filtration rate; HDL-C, high density lipoprotein cholesterol; HT, hormone therapy; MESA, Multi-Ethnic Study of Atherosclerosis; MET, metabolic equivalent of task; SHBG, sex hormone binding globulin; SAT, subcutaneous adipose tissue; T, testosterone; VAT, visceral adipose tissue.

Data are presented as mean (SD) or median (IQR) or frequency (percentage).

*Data were collected only in women.

### Sex hormones and adipokines in women

Table 2 shows the multivariable-adjusted association between sex hormones and adipokines (leptin, resistin, and adiponectin) in post-menopausal women.

**TABLE 2:** Multivariable-adjusted association between sex hormones and adipokines in women.

Percent difference (95% CI)				
	Model 1, *n* = 850	Model 2a, *n* = 850	Model 2b, *n* = 668	Model 3, *n* = 668
**Leptin**				
Total T	**9 (2, 15)**	0 (−4, 5)	−2 (−7, 3)	0 (−5, 5)
Free T	**26 (19, 34)**	6 (0, 12)	−1 (−7, 5)	0 (−7, 6)
Bio T	**19 (12, 26)**	2 (−3, 7)	−3 (−8, 3)	−2 (−7, 4)
E2	**10 (3, 17)**	**7 (1, 14)**	4 (−2, 11)	3 (−3, 10)
DHEA	5 (−1, 11)	0 (−5, 5)	−3 (−8, 2)	−1 (−6, 5)
SHBG	−**21 (**−**26,**−**17)**	−**6 (**−**11, 0)**	1 (−5, 8)	0 (−6, 7)
T/E2	−3 (−8, 3)	−5 (−10, 1)	−5 (−10, 1)	−3 (−8, 3)
**Resistin**				
Total T	2 (0, 5)	1 (−1, 4)	2 (−1, 5)	2 (−2, 5)
Free T	2 (−1, 5)	−1 (−4, 3)	0 (−4, 4)	−1 (−5, 3)
Bio T	2 (−1, 5)	0 (−3, 3)	1 (−3, 5)	1 (−3, 4)
E2	0 (−3, 3)	−2 (−5, 2)	−2 (−5, 2)	−2 (−5, 2)
DHEA	−1 (−3, 2)	−1 (−4, 2)	−2 (−5, 2)	−1 (−4, 2)
SHBG	−2 (−4, 1)	1 (−3, 4)	1 (−3, 5)	1 (−3, 5)
T/E2	1 (−1, 4)	2 (−1, 6)	3 (−1, 7)	3 (−1, 6)
**Adiponectin**				
Total T	−**5 (**−**8,**−**2)**	−3 (−7, 0)	0 (−3, 4)	1 (−3, 5)
Free T	−**16 (**−**19,**−**13)**	−**18 (**−**21,**−**14)**	−**14 (**−**18,**−**10)**	−**11 (**−**16,**−**7)**
Bio T	−**12 (**−**16,**−**9)**	−**11 (**−**15,**−**8)**	−**7 (**−**11,**−**3)**	−**5 (**−**9, 0)**
E2	−**6 (**−**10,**−**3)**	−**7 (**−**11,**−**2)**	−**5 (**−**10,**−**1)**	−**7 (**−**11,**−**2)**
DHEA	−**6 (**−**9,**−**2)**	−**5 (**−**9,**−**1)**	−2 (−6, 2)	−2 (−6, 2)
SHBG	**19 (15, 24)**	**22 (17, 27)**	**18 (12, 23)**	**14 (8, 20)**
T/E2	2 (−1, 6)	3 (−2, 7)	5 (0, 10)	**6 (1, 11)**

Bio, Bioavailable; E2, Estrogen; DHEA, Dehydroepiandrosterone; SHBG, Sex hormone binding globulin; T, Testosterone.

Logarithmically transformed adipokines and one SD of logarithmically transformed sex hormones were used for the analysis.

Percent difference was calculated from ([Exp (β) −1]*100) derived from linear regression models.

Statistically significant results at *p* < 0.05 are in bold font.

If 0 value is bolded in table, this is because with additional digits, the value excluded 0.

Model 1: age, race/ethnicity, and MESA field site. Model 2a: model 1 covariates plus smoking, BMI, education, physical activity, and current use of hormone therapy.

Model 2b: model 2a covariates plus subcutaneous adipose tissue and visceral adipose tissue.

Model 3: model 2b covariates plus total cholesterol, HDL-C, use of lipid-lowering medication, systolic blood pressure, use of anti-hypertensive medication, diabetes, and eGFR. Premenopausal women were excluded from the analysis.

Sample sizes for Bio T: models 1 and 2a = 845, models 2b and 3 = 663.

#### Leptin

When adjusting for age, race/ethnicity, and MESA field site (model 1) we found that total T [percent difference 9% (95% CI, 2, 15)], free T [26% (19, 34)], bio T [19% (12, 26)], and E2 [10% (3, 17)] all had positive associations with leptin levels, while SHBG [−21% (−26, −17)] had an inverse association. However, after further adjusting for smoking, BMI, education, physical activity, and current use of hormone therapy (model 2a), only the positive association of E2 [7% (1, 14)], and the inverse association of SHBG [−6% (−11, 0)], with leptin remained statistically significant. Additional adjustment for CVD risk factors further attenuated these associations which became no longer statistically significant.

#### Resistin

There were no significant associations between the sex hormones and resistin for post-menopausal women.

#### Adiponectin

Free T, bio T, and E2 had a significant inverse association with adiponectin across all models. In the fully adjusted model for CVD risk factors (model 3), the relationship with lower adiponectin was as follows: free T [−11% (−16, −7)], bio T [−5% (−9, 0)], and E2 [−7% (−11, −2)]. On the other hand, SHBG was positively associated with adiponectin, even after full covariate adjustment for CVD risk factors in model 3 [14% (8, 20)]. Although free T was associated with lower adiponectin, the T/E2 ratio was associated with higher adiponectin level [6% (1, 11)].

### Sex hormones and adipokines in men

Table 3 shows the multivariable-adjusted association between sex hormones and adipokines in men.

**TABLE 3:** Multivariable-adjusted association between sex hormones and adipokines in men.

Percent difference (95% CI)				
	Model 1, *n* = 961	Model 2a, *n* = 961	Model 2b, *n* = 668	Model 3, *n* = 668
**Leptin**				
Total T	−**20 (**−**25,**−**15)**	−**8 (**−**13,**−**3)**	−**8 (**−**13,**−**2)**	−**8 (**−**14,**−**2)**
Free T	**21 (13, 31)**	**6 (0, 13)**	**8 (1, 15)**	**8 (1, 15)**
Bio T	−**13 (**−**9,**−**7)**	−5 (−10, 0)	−4 (−10, 3)	−5 (−11, 2)
E2	**7 (1, 15)**	2 (−4, 7)	3 (−2, 8)	3 (−2, 9)
DHEA	−**12 (**−**18,**−**5)**	−**8 (**−**13,**−**2)**	−6 (−11, 0)	−5 (−11, 1)
SHBG	−**20 (**−**26,**−**15)**	−**6 (**−**12,**−**1)**	−**8 (**−**13,**−**2)**	−**8 (**−**13,**−**2)**
T/E2	−**22 (**−**27,**−**17)**	−**9 (**−**13,**−**3)**	−**8 (**−**13,**−**3)**	−**9 (**−**14,**−**3)**
**Resistin**				
Total T	−**3 (**−**6,**−**1)**	−**3 (**−**5, 0)**	−**5 (**−**8,**−**1)**	−**5 (**−**8,**−**1)**
Free T	−1 (−3, 2)	−1 (−4, 2)	0 (−4, 3)	−3 (−6, 1)
Bio T	−**4 (**−**6,**−**1)**	−**3 (**−**6,**−**1)**	−**5 (**−**9,**−**2)**	−**6 (**−**9,**−**3)**
E2	1 (−1, 4)	1 (−2, 4)	1 (−2, 4)	0 (−3, 3)
DHEA	0 (−3, 3)	0 (−3, 3)	1 (−3, 4)	1 (−3, 4)
SHBG	0 (−2, 3)	1 (−2, 4)	0 (−4, 3)	2 (−2, 5)
T/E2	−**4 (**−**6,**−**2)**	−**3 (**−**6,**−**1)**	−**4 (**−**7,**−**1)**	−**3 (**−**6, 0)**
**Adiponectin**				
Total T	**7 (4, 11)**	**5 (2, 9)**	**8 (3, 13)**	**6 (2, 11)**
Free T	−**11 (**−**14,**−**8)**	−**10 (**−**13,**−**6)**	−**8 (**−**12,**−**4)**	−**7 (**−**11,**−**2)**
Bio T	2 (−1, 6)	1 (−3, 4)	3 (−2, 7)	2 (−2, 7)
E2	−3 (−6, 0)	−2 (−5, 1)	−1 (−5, 2)	−1 (−5, 2)
DHEA	−2 (−2, 6)	1 (−3, 5)	2 (−2, 7)	2 (−2, 6)
SHBG	**13 (9, 17)**	**11 (7, 15)**	**9 (4, 14)**	**7 (3, 12)**
T/E2	**9 (5, 12)**	**7 (3, 10)**	**7 (3, 11)**	**6 (2, 10)**

Bio, Bioavailable; E2, Estrogen; DHEA, Dehydroepiandrosterone; SHBG, Sex hormone binding globulin; T, Testosterone. Logarithmically transformed adipokines and one SD of logarithmically transformed sex hormones were used for the analysis.

Percent difference was calculated from ([Exp (β) −1]*100) derived from linear regression models. Statistically significant results at *p* < 0.05 are in bold font.

Model 1: age, race/ethnicity, and MESA field site.

Model 2a: model 1 covariates plus smoking, BMI, education, and physical activity. Model 2b: model 2a covariates plus subcutaneous adipose tissue and visceral adipose tissue.

Model 3: model 2b covariates plus total cholesterol, HDL-C, use of lipid-lowering medication, systolic blood pressure, use of anti-hypertensive medication, diabetes, and eGFR.

#### Leptin

Total T [−8% (−14, −2)], SHBG [−8% (−13, −2)], and T/E2 ratio [−9% (−14, −3)] were significantly inversely associated with leptin in men even in the fully adjusted model (model 3), adjusted for CVD risk factors. A positive relationship between free T [8% (1, 15)] and leptin was also statistically significant in model 3. Bio T was inversely associated with leptin in demographic adjusted model, but not in further adjusted models.

#### Resistin

Total T [−5% (−8, −1)], bio T [−6% (−9, −3)], and T/E2 ratio [−3% (−6, 0)] had an inverse relationship with resistin in the fully adjusted model (model 3).

#### Adiponectin

Total T [6% (2, 11)], SHBG [7% (3, 12)], and T/E2 ratio [6% (2, 20)] had a positive relationship with adiponectin, while free T [−7% (−11, −2)] had an inverse relationship in the fully adjusted model (model 3), adjusted for CVD risk factors.

### Interaction testing

In our supplemental analysis, we found significant interactions in women between obesity status and sex hormone levels (*p* < 0.05 for leptin, resistin, and adiponectin) (Supplementary Table 1). Among women with a BMI < 30 kg/m^2^, free T [20% (11, 30)] and bio T [15% (6, 24)] were significantly positively associated with leptin when adjusting for age, race/ethnicity, field site, smoking status, education, physical activity, and current use of hormone therapy (model 2a). This association was attenuated in models further adjusted for CVD risk factors. In this BMI < 30 kg/m^2^ group, there was also a significant positive association between total T [4% (0, 8)] and resistin across models 1, 2a, and 2b. Lastly, for post-menopausal women with a BMI ≥ 30 kg/m^2^ there is a significant inverse association between total T [−7% (−13, −1)] and adiponectin across models 1 and 2a. There was no significant interaction between obesity status and sex hormone levels in men.

## Discussion

### Overall summary of finding

Adipokines have been implicated in cardiometabolic diseases, with higher leptin and resistin levels being associated with elevated CVD risk (33, 42), and adiponectin showing favorable associations (31). However, the relationship of sex hormones with adipokine levels had not been well established previously. In this cross-sectional study from a multi-ethnic cohort, we found mixed associations of sex hormones with adipokine levels. In both post-menopausal women and men, a more androgenic sex profile (characterized by higher free T and lower SHBG) was significantly associated with lower adiponectin, a cardio-protective adipokine, even after adjusting for other CVD risk factors. But unexpectedly, a higher T/E2 ratio was associated with higher adiponectin levels in both sexes. Additionally in men, higher free T was associated with higher leptin levels, whereas higher total T, SHBG, and T/E2 were associated with lower leptin levels. Also in men higher total T, bio T, and T/E2 were associated with lower resistin levels. No association was seen between sex hormones with leptin and resistin levels in women, after full covariate adjustment.

### Sex hormones and adipokines in women

Prior MESA studies have suggested that a more androgenic sex hormone pattern (with higher free T levels) is associated with an adverse cardiometabolic profile and increased CVD risk in post-menopausal women (6–10). Women with higher androgen levels have more visceral fat (11); thus we had hypothesized that a more androgenic sex hormone profile would be associated with an adverse adipokine profile in postmenopausal women. Some of our findings were consistent with this hypothesis. At least in a limited adjusted model, higher androgen levels (i.e., free T and bio T) were associated with higher leptin levels in women, although this was no longer significant after adjusting for other CVD risk factors. In our supplemental analysis, this positive association between free T and leptin appeared stronger for women with a BMI < 30 compared to ≥ 30 kg/m^2^. We did not find any relationship of sex hormones with resistin in women.

Also consistent with our hypothesis, greater androgens such as free T and bio T were associated with lower levels of adiponectin, a favorable adipokine, in women. On the other hand, surprisingly and contrary to our hypothesis, higher total T/E2 ratio and lower E2 levels were positively associated with higher adiponectin. Although estrogen is thought to be cardioprotective in pre-menopausal women, its role is further complicated in our population of post-menopausal women. After menopause, endogenous E2 levels are low in women not on hormone therapy, even lower than in men. Circulating E2 levels in post-menopausal women, as well as in men, reflect peripheral conversion of testosterone to estradiol, which may be why the E2 results track with the results of free T. Interactions of E2 with adipokines in conferring cardiovascular risk or protection remain complicated and warrant further study. Total T includes both T in the bound and unbound states, and higher SHBG was also positively associated with adiponectin. It may be that free (unbound) T is a better marker of CVD risk in women. In our supplemental analysis, the inverse relationship between free T and adiponectin was stronger for post-menopausal women with BMI ≥ 30 kg/m^2^, compared to women with BMI < 30. Overall, taken together, our results further suggest that a more androgenic sex profile is inversely associated with an adipokine thought to be protective against cardiovascular risk in women.

### Sex hormones and adipokines in men

Prior studies have suggested that a low androgen (hypogonadal state) is associated with visceral adiposity (11) and CVD risk (15, 16) in men, so we had hypothesized that higher androgens in men would be associated with a favorable adipokine profile. For men, higher androgen levels in the form of total T and T/E2 ratio, as well as lower SHBG levels, were associated with lower leptin levels, even after adjustment for other CVD risk factors. Higher androgen levels in the form of total T, bio T, and T/E2 ratio were also associated with lower resistin levels. Higher androgen levels in the form of total T and T/E2 were also associated with higher adiponectin levels, a cardio-protective adipokine. These results were consistent with our hypothesis of androgens being associated with a favorable adipokine profile in men.

However, not all findings aligned with our *a priori* hypothesis. Unexpectedly, higher free T was positively associated with leptin and inversely associated with adiponectin in men—an unfavorable adipokine pattern. It should be noted that approximately 68% of total T in circulation is tightly bound to SHBG and is biologically inactive in this form. The biologically active forms of T (i.e., bio T) consists of the ∼30% of T which is loosely bound to albumin and the 2% circulating as free T (43). In our analyses for men, the associations of free T were generally in the opposite directions of total T and SHBG. It may be that free T is a better estimate of available androgens in men and reflect the true relationship with cardiometabolic risk. This discordance highlights the complexity of understanding sex hormone patterns, when looking at a single hormone level. But generally the associations of free T with lower adiponectin levels mirrored that seen in women and further support the potentially adverse effects of endogenous androgens and the development of CVD in both sexes.

### Putting findings into clinical context

We examined the association of sex hormones with adipokines, because a more unfavorable fat-distribution and adipokine profile may be an intermediary step linking an androgenic sex hormone profile with CVD risk. There are multiple proposed mechanisms by which adipokines influence CVD risk. Adipokines have been shown to influence chronic inflammation and metabolic disorders, including glucose intolerance, hyperlipidemia, and arterial hypertension (29, 44, 45), and vascular calcification (42, 46). Both estrogens and androgens have been implicated in the modulation of adipokines (47–50). Postmenopausal women, who have low estradiol levels, have increased central adiposity, increased leptin levels, and decreased adiponectin levels (49). Our findings suggest that a more androgenic sex hormone state after menopause is related to this unfavorable adipokine pattern. Reproductive age women with polycystic ovary syndrome also have hyperandrogenic sex hormone profiles that were associated with higher levels of leptin and lower levels of adiponectin (47, 48, 51). Although we studied post-menopausal women in our analysis, our findings are generally consistent with this findings by demonstrating higher free T and lower SHBG being associated with lower adiponectin.

Despite the associations of adipokines with cardiometabolic risk factors, the association of adipokines with future CVD events has been inconsistent, with both positive and negative (null) associations (32, 33, 52–54). In prior work from MESA, the association of leptin with incident CVD events was attenuated after accounting for BMI and traditional CVD risk factors (32), whereas greater resistin levels remained independently associated with CVD even after accounting for these factors (33). This underscores the complexity of our understanding of the pathways adipokines play in CVD risk.

### Strengths and limitations

Our study has significant strengths. Prior studies from the MESA cohort have previously examined the independent associations between sex hormones and CVD risk (6) as well as adipokines and CVD risk (32, 33). We now newly examine the relationship of sex hormones and adipokines with each other. Our findings generally suggest there may be a link between more androgenic sex hormone profiles (captured by free T levels) and the adipokines that are thought to increase CVD risk, and a negative association with adipokines thought to be cardioprotective. Some findings though were inconsistent with our hypothesis. Although the clinical significance of our findings remains uncertain, this work suggests a possible pathophysiologic relationship between adipose-derived biomarkers and sex hormones, leading to differences in cardiovascular risk between the sexes. Little is known about this interaction and we provide data aimed at filling this need and hope future studies can provide additional clarity.

However, our study also has a number of limitations. Sex hormone levels and adipokines were only measured once in the MESA cohort, so we could not examine changes in these levels over time. Furthermore, we use a cross-sectional design and the sex hormones and adipokine levels were measured at different periods in time. Although we adjusted for confounding variables, residual confounders may still be present. Finally, we performed multiple analyses therefore some of our results may have been due to chance. However, the aim of our study was to be exploratory in nature with associations found to be further hypothesis-generating. Future studies should seek to understand mechanisms linking sex hormones, adipokines, and CVD risk.

## Conclusion

In summary, we found in a diverse community-based cohort that an androgenic sex hormone profiles, characterized by higher free T and lower SHBG levels, were independently associated with the risk-enhancing adipokine of leptin and inversely associated with the more cardioprotective adipokine of adiponectin in both men and post-menopausal women. These findings may provide mechanistic insight in the interplay between sex hormones, adipokines, and CVD risk.

## Data availability statement

The datasets presented in this article are not readily available because the data, methods, and materials used to conduct this study will be made available to other researchers for the purposes of reproducing or expanding on the results upon application to and approval by the MESA publications and presentations committee. Requests for the use of MESA data can also be done through the National Heart, Lung, and Blood Institute Biologic Specimen and Data Repository Coordinating Center (https://biolincc.nhlbi.nih.gov/studies/mesa/). Requests to access the datasets should be directed to chsccweb@u.washington.edu.

## Ethics statement

The studies involving human participants were reviewed and approved by the Johns Hopkins School of Medicine Institutional Review Board, IRB3 committee. The patients/participants provided their written informed consent to participate in this study.

## Author contributions

BV and EM designed the study and wrote the initial draft. OOg performed the statistical analysis. MA secured grant funding for the measurement of adipokines and body composition. CN, BK, CR, OOs, MA, and AB provided critical revisions for important intellectual content. All authors approved the final draft for submission.
