# Supplementary material for: Associations between endogenous sex hormone levels and adipokine levels in the Multi-Ethnic Study of Atherosclerosis
Source: Front Cardiovasc Med. 2023 Jan 13;9:1062460. doi: 10.3389/fcvm.2022.1062460 (PMC9880051; doi:10.3389/fcvm.2022.1062460)
Supplement: Supplementary file 1 [file Table_1.DOCX]

**SUPPLEMENTAL MATERIAL**

| Table S1. Multivariable-adjusted association between sex hormones and adipokines in women stratified by BMI <30 and ≥ 30kg/m^2^ | | | | |
| --- | --- | --- | --- | --- |
| Percent difference (95% CI) | | | | |
|  | Model 1 | Model 2a | Model 2b | Model 3 |
| *Leptin: <30kg/m^2^* |  |  |  |  |
| Total T | 7 (0, 14) | 7 (0, 15) | 3 (-3, 9) | 4 (-2, 10) |
| Free T | **18 (10, 27)** | **20 (11, 30)** | -3 (-9, 4) | -1 (-8, 7) |
| Bio T | **13 (6, 21)** | **15 (6, 24)** | 1 (-6, 7) | 2 (-5, 9) |
| *Leptin: ≥30kg/m^2^* |  |  |  |  |
| Total T | -4 (-11, 3) | -4 (-11, 3) | **-12 (-20, -3)** | -7 (-15, 3) |
| Free T | 0 (-8, 8) | 3 (-6, 14) | 2 (-9, 14) | 1 (-10, 13) |
| Bio T | -4 (-10, 4) | -3 (-10, 5) | -9 (-18, 1) | -5 (-15, 5) |
| *Resistin: <30kg/m^2^* |  |  |  |  |
| Total T | **4 (0, 7)** | **4 (0, 7)** | **4 (0, 8)** | 4 (0, 7) |
| *Resistin: ≥30kg/m^2^* |  |  |  |  |
| Total T | -3 (-7, 2) | -3 (-7, 2) | -4 (-11, 3) | -3 (-10, 4) |
| *Adiponectin: <30kg/m^2^* |  |  |  |  |
| Total T | -2 (-6, 3) | -2 (-6, 2) | 0 (-5, 4) | 1 (-3, 5) |
| *Adiponectin: ≥30kg/m^2^* |  |  |  |  |
| Total T | **-8 (-14, -2)** | **-7 (-13, -1)** | 2 (-6, 12) | 1 (-8, 11) |
| Abbreviations: Bio, Bioavailable; T, Testosterone.  Logarithmically transformed adipokines and one SD of logarithmically transformed sex hormones were used for the analysis.  Percent difference was calculated from ([Exp (β) -1]*100) derived from linear regression models.  Statistically significant results at p<0.05 are in bold font.  Model 1: age, race/ethnicity and MESA field site.  Model 2a: model 1 covariates plus smoking, education, physical activity and current use of hormone therapy.  Model 2b: model 2a covariates plus subcutaneous adipose tissue and visceral adipose tissue.  Model 3: model 2b covariates plus total cholesterol, HDL-C, use of lipid-lowering medication, systolic blood pressure, use of anti-hypertensive medication, diabetes and eGFR.  Premenopausal women were excluded from the analysis.  P values for interaction by BMI  **Leptin:**  *Total T: 0.032*  *Free T: 0.037*  *Bio T: 0.017*  **Resistin:**  *Total T: 0.042*  **Adiponectin:**  *Total T: 0.047* | | | | |
